# 3D Bioprinting and In Vitro Cardiovascular Tissue Modeling

**DOI:** 10.3390/bioengineering4030071

**Published:** 2017-08-18

**Authors:** Jinah Jang

**Affiliations:** Department of Creative IT Engineering, Pohang University of Science and Technology (POSTECH), Pohang 37673, Kyungbuk, Korea; jinahjang@postech.ac.kr; Tel.: +82-54-279-8821

**Keywords:** bioprinting, 3D tissue modeling, biomaterials, stem cell, cardiovascular tissue

## Abstract

Numerous microfabrication approaches have been developed to recapitulate morphologically and functionally organized tissue microarchitectures in vitro; however, the technical and operational limitations remain to be overcome. 3D printing technology facilitates the building of a construct containing biomaterials and cells in desired organizations and shapes that have physiologically relevant geometry, complexity, and micro-environmental cues. The selection of biomaterials for 3D printing is considered one of the most critical factors to achieve tissue function. It has been reported that some printable biomaterials, having extracellular matrix-like intrinsic microenvironment factors, were capable of regulating stem cell fate and phenotype. In particular, this technology can control the spatial positions of cells, and provide topological, chemical, and complex cues, allowing neovascularization and maturation in the engineered cardiovascular tissues. This review will delineate the state-of-the-art 3D bioprinting techniques in the field of cardiovascular tissue engineering and their applications in translational medicine. In addition, this review will describe 3D printing-based pre-vascularization technologies correlated with implementing blood perfusion throughout the engineered tissue equivalent. The described engineering method may offer a unique approach that results in the physiological mimicry of human cardiovascular tissues to aid in drug development and therapeutic approaches.

## 1. Introduction

Cardiovascular diseases (CVDs) are chronic illnesses and the leading cause of morbidity and mortality among the high-income countries of the world, accounting for more than one-third of total deaths. In particular, myocardial infarction (MI) is a predominant reason for heart failure [1]. The blockage of the coronary artery gives rise to the hypoxia and death of cardiomyocytes and severe inflammation, which results in the degeneration of the left ventricular extracellular matrix and scar tissue formation. This phenomenon leads to the dilation of the left ventricle and reduces cardiac contractility. However, cardiomyocytes are barely repopulated in adults at the infarcted area. The available strategies for end-stage ischemic heart failure are heart transplantation, coronary artery bypass grafting (CABG), and the use of a left ventricular assist device (LVAD); however, transplantation is hampered by the severe donor shortage and the problem of immune rejection after transplantation [2]. These reasons have led researchers to seek alternatives to meet the currently unmet clinical needs.

Stem cell therapy is arising as an alternative therapeutic approach to overcome the current therapeutic limitation [3,4]. Over the last decade, there have been numerous clinical trials with adult stem cells, particularly mesenchymal stem cells, to take advantage of their self-healing, multipotency, immune-privilege, and regeneration capabilities [5,6]. Autologous bone marrow-derived cells (e.g., mononuclear cells selected by surface markers such as CD34) [7,8], cardiac stem (progenitor) cells (e.g., isolated using surface markers including Sca1, c-kit, and Isl) [9,10,11], and cardiosphere-derived cells [12,13] have been widely applied in clinical trials. Autologous skeletal myoblasts have also been used for developing cell sheet applications [14,15,16]. However, according to a recent meta-analysis, these therapeutics have not been so successful. This is because the current approaches of engrafting cells into patients’ damaged myocardium (e.g., intracoronary artery or intramyocardial delivery) might not provide a good environment for surviving, and there could be a lot of variance on the quality of stem cells and clinical conditions depending on patients [17]. In addition, these approaches have shown less stem cell homing efficacy, and it has been difficult for the injected cells to survive in the hostile microenvironment because of the low vasculature, hardened tissues caused by scars, and inflammation processes. The poor survival rate has been attributed to continued ischemia within the graft and surrounding tissues.

Now, the generation of human pluripotent stem cell (hPSC)-derived cardiovascular cells is of growing interest for multiple applications [18,19,20]. Many researchers have developed human embryonic stem cells (ESCs) or induced pluripotent stem cell (iPSCs)-based directed differentiation protocols into various cardiovascular cells, including cardiomyocytes [21,22], endothelial cells [23], smooth muscle cells [24], epicardial cells [25], and even Purkinje neurons [26] and pacemaker cells [27]. In the long term, these cell sources are expected to be implanted directly into patients, yet there are still several concerns about the moral implications of using human embryos and the genetic modification and alteration process of using iPSCs. With the use of the abovementioned cell sources, it is recommended to assess an in vitro testing model of human development or a disease model to understand underlying mechanism. The patient-derived iPSCs with genetically inherent cardiac diseases can provide a remarkable opportunity to study disease mechanisms [28,29,30]. In addition, there are fundamental therapeutics based on the engineered cardiovascular tissues, which can be achieved by the convergence of the various advanced techniques, including tissue engineering, biomaterials, microfabrication, and stem cell engineering. It could also be of particular interest to pharmaceutical companies to create a new cardioactive compound and reduce the cardiotoxicity, such as drug-induced QT prolongation [31,32].

The lack of control over the organization of the vasculature hampers the function of tissue-engineered fillers or constructs. In this regard, recent studies suggest that the incorporation of cells (e.g., endothelial cells, hematopoietic stem cells, mesenchymal stem cells (MSCs), adipose-derived stem cells (ASCs), and dental pulp stem cells (DPSCs)) or biofactors (e.g., VEGF, FGF, HGF, and Ang-1) can accelerate the vascularization of engineered constructs as well as improve long-term tissue survival [33,34]. However, it is difficult for randomly distributed capillaries to supply oxygen and nutrients to the constructs, which makes it complicated to recapitulate the vasculature of organs that are comprised of multiple cells and complex networks. In this regard, the patterning of vascular cell sources can play an important role in rapid vascularization and the maturation of the engineered tissue. Consequently, new approaches applying microfabrication technology (e.g., photolithography-based tissue molding technique [35], micropatterning-based endothelial cell alignment [36,37], bioprinting-based multiple cellular deposition [38,39,40]) can control the spatial positions of cells, allowing endothelial tubulogenesis (vasculogenesis) in the engineered construct [41]. Although the 3D bioprinting-based tissue modeling method is still in its early stages, it is considered a promising approach to produce a desired porous inner structure so that oxygen and nutrients can be easily supplied. In addition, this can place multiple types of cells depending on the tissue layout and make precisely controlled 3D tissues by recapitulating the original complexity, geometry, and compositions of target tissues.

Therefore, this review focuses on the state of the art of 3D bioprinting techniques in the field of cardiovascular tissue engineering and their future applications in in vitro testing platforms to defeat CVDs.

## 2. Printing Techniques for 3D Tissue Fabrication

The important features (e.g., position, size, shape, number of cells, and physiological complexity) need to be recapitulated to engineer human tissues [42]. In particular, the patterning of biological components via 3D bioprinting can be integral to building tissue models [43,44], and the signaling pathways, force generation, and transmission (e.g., body fluid flow, interactions among cells and microenvironments, extrinsic mechanical forces) are critical to the self-organization phenomena driving tissue morphogenesis [45,46]. In particular, this technology can control the spatial positions of cells, and provide topological, chemical, and complex cues, allowing neovascularization and maturation in the engineered cardiovascular tissues. To achieve structural/functional performance of target tissues, several important techniques need to be considered: Printable biomaterials (bioink), advanced bioprinting techniques, and micro-environmental regulations for promoting tissue morphogenesis.

### 2.1. Bioinks

Bioinks need to possess several important characteristics, such as biocompatibility, printability, mechanical and structural integrity, biomimicry, and biodegradability. There are a number of reviews on the required printability, structural integrity and rheological characteristics of bioinks which exceed the scope of this review. If you would like to get further information, I recommend the reviews by Jos Malda [47] and Bin Duan [48].

The two different categories of widely used bioinks, natural and synthetic polymers, have strikingly definitive advantages and disadvantages. The natural polymer-based bioinks are typically isolated from natural sources and have been used for improving the biological features of printed constructs. This provides tissue-specific biochemical and physical stimuli to guide cellular behaviors (e.g., migration, proliferation, differentiation, and maturation). It also induces matrix-driven neo tissue formation by communicating with cells and matrices, yet natural polymers have less mechanical stability, higher variations in molecular weight and structure from batch to batch than synthetic polymers, and the potential risk of pathogen transfer from the originating organism [49]. Natural polymers can be classified according to their composition as proteins (e.g., collagen [50], gelatin [51], fibrin [52], and silk [53]), polysaccharides (e.g., alginate [54] and chitosan [55]), or glycosaminoglycans (e.g., hyaluronic acid [56]).

On the other hand, synthetic polymers provide a mechanically robust structure; however, the lack of active binding sites, which can induce the cellular signaling pathway, hampers the adhesion of cells and can even result in cell death. Thus, a limited number of synthetic polymers is used for directly encapsulating cells for printing processes (e.g., polyethylene glycol-diacrylate (PEG-DA) [57] and PEG [58]), and they have controllable mechanical strength and degradation capability. In addition, the chemical conjugation of synthetic and natural polymers, such as gelatin-methacrylate (GelMA) [59] or PEGylated gelatin methacrylate (PEGgelMA) [60], may achieve a biologically advanced bioink system. The various thermoplastic polymers (e.g., PCL [61] and PLGA [62]) are utilized for making polymeric frameworks or fugitive (sacrificial) polymers (e.g., Pluronic F127 [59] and poly vinyl alcohol (PVA) [63]).

### 2.2. Bioprinting Techniques

#### 2.2.1. Microextrusion-Based Bioprinting Systems

**Figure 1:**
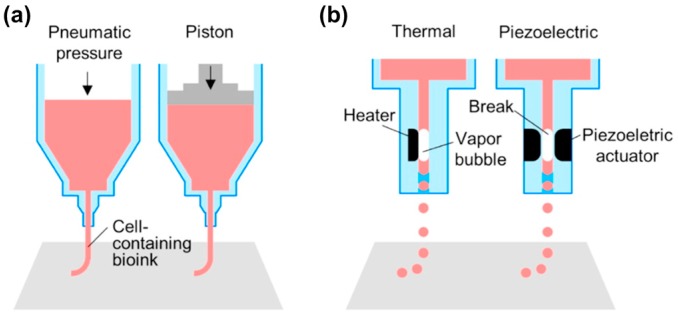

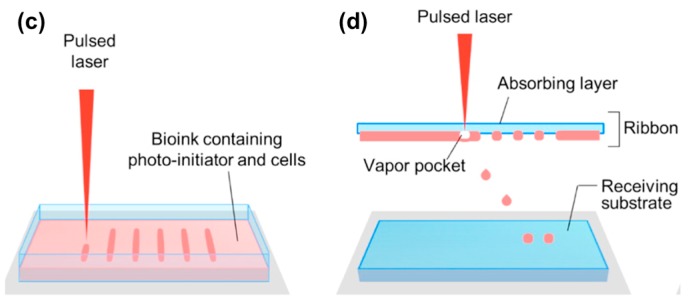
Schematic illustrations of: (**a**) microextrusion; (**b**) ink-jet; (**c**) stereolithography; and (**d**) laser-assisted printing. Reproduction with permission from [73].

The microextrusion technique is a precise computer-controlled dispensing system that moves together with the multiple degrees of freedom motion stages (Figure 1a). Metal or plastic syringes are used to contain materials that are dispensed by pneumatic pressure, screw-driven, or robotic-driven forces. The size and structure of the nozzles placed at the end of the syringe are selected according to the specification of the structural design. For example, co-axial or multiple axial nozzles have the potential to be applied to the engineering of blood vessels or nerve conduits due to their capability of directly fabricating perfusable tube structures [64,65]. The materials loaded in the syringe can be maintained in melt-flowable or liquid status by heating or cooling through the temperature controlling system. To implement the automatic manufacturing process that involves every part of the microextrusion system, it is usually connected with the electrical units and operated by computer-based code. This system can create 3D printed tissues by stacking 2D patterns of the desired shape and cellular organization through a layer-by-layer process. The dispensing volume from the syringe is regulated by adjusting the dispensing rate (e.g., by changing the level of pneumatic pressure, speed of plunger displacement, or screw rotation), the velocity of the dispensing head, and the size of the nozzles. This technique can particularly provide more tissue microenvironment to the engineered construct because the cells are directly encapsulated in the extracellular matrix (ECM)-like bioink during and after the processes. In addition, it has a wider versatility of material selection.

Recently, bioprinters equipped with multiple extruders have been developed to utilize multiple bioinks with different cell types, which have enabled the creation of physiologically relevant heterogeneous 3D tissues. In addition, a ultraviolet (UV) light-based curing system and vision system to set the origin are installed to support the function of the printer [62]. Although this system provides relatively lower resolution (~100 μm) compared to the other methods, this technique has a wider range of applied viscosities from 30 to 6 × 10^7^ mPa·s, which allows various types of bioinks (e.g., both synthetic and natural polymers) to be adapted in the same system.

#### 2.2.2. Ink-Jet Bioprinting Systems

Ink-jet printing has a high-resolution characteristic because it can generate very small droplets (1–100 picolitres) by using a mechanical pulse of the printing head (Figure 1b) [66]. This system requires lower viscose bioink (3.5–12 mPa·s) than other techniques. Thermal and piezo-electric methods are commonly used to shoot the bioink from the head, and the motion stage facilitate to generate specific 2D patterns or 3D structures. In particular, a thermal inkjet printer makes drops of bioinks by a heating pulse from the micro-heater [67]. A piezoelectric inkjet printer, the direct mechanical pulse is generated by a piezoelectric actuator and causes a force to expel bioink droplets from the printing head [68]. The diameter of the droplet usually depends on the size of nozzles, and the currently used diameter is equal to or larger than 30 μm [69].

#### 2.2.3. Laser-Assisted Bioprinting Systems

Stereolithography (SLA) and laser-induced forward transfer (LIFT) are the predominant techniques of the laser-assisted bioprinting system. SLA is the oldest 3D printing technology that can create 3D structures from polymerized photopolymers by exposing the pre-designed patterns to highly accurate optics using focused light sources, including UV, infrared, and visible light (Figure 1c) [70]. This system is composed of the liquid photopolymer reservoir, light source, and three-axis motion stage. It uses single- or two-photon absorption, which can be defined by the selection of laser sources. In single-photon absorption, the pattern can be exposed in two different ways: (1) beam scanning; and (2) image projection. The image projection method can significantly reduce the time for printing and is much simpler compared to beam scanning. The two-photon absorption system uses a femtosecond laser to implement two-photon excitation, and photopolymerization is induced in a very precise region without affecting the other side of the polymer reservoir.

The LIFT method produces a cell-containing droplet by pulsing onto a cell-containing bioink layer (called ribbon), which is placed below a laser energy-absorbing layer (Figure 1d) [71,72]. This “aim-and-shoot” procedure induces localized evaporation of the absorbing layer, and the generated gas pressure pushes the bioink droplet off the ribbon toward the hydrogel-coated collector slide. This nozzle-free system often uses highly viscous bioinks (1–300 mPa·s) to create a robust 3D structure composed of a sophisticated pattern of cells, and it offers the highest resolution to control the droplets.

## 3. 3D Modeling of Cardiovascular Tissues

Mechanical, chemical, and topological cues have far-reaching implications for the responses of engineered tissues. The many biofabrication techniques have shown the potential to improve the viability, intrinsic functions, and electrophysiological features of cardiomyocytes and the other cardiac cells (e.g., endothelial cells, smooth muscle cells, and fibroblasts). In particular, hierarchical design is a critical factor in higher order tissue modeling, which is related to the proper selection of cells, bioinks, and external stimuli and the recapitulation of natural hemodynamic conditions and vascular networks.

### 3.1. The Need for 3D Models

With the 2D culture of cells on flat surfaces, it is difficult to understand their original functions inherently designed for 3D existence [74,75]. Despite its valuable contributions to biomedical research, it is difficult to recapitulate the complex cell–cell interactions and their functions or accurately predict the in vivo efficacy of drugs with 2D culture. To overcome these limitations, many researchers have prompted the development of more complex culture platforms in 2D surfaces, including micro/nanopatterns [76], pillars [77], surface modification [78], and cellular patterning [79]. In addition, multiple cell types (e.g., endothelial, cardiac fibroblast, and other stromal cell types) can be incorporated to provide paracrine signaling among the cardiovascular cell sources or support normal physiological conditions and the maturation of cardiomyocytes.

**Figure 2:**
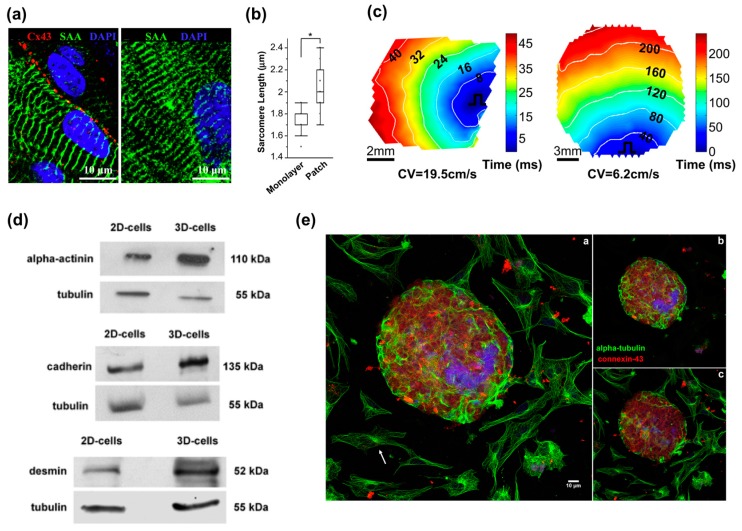
3D models for cardiovascular tissue modeling: (**a**) Representative immunostainings of hESC-CMs in a two-week-old tissue patch coupled by connexin-43 gap junctions (left) and in two-week-old monolayers (right); (**b**) Median and quartile sarcomere lengths in monolayers (*n* = 58 hESC-CMs) and patches (*n* = 106 hESC-CMs). * *p* < 0.0001; (**c**) Representative isochrone maps during 1 Hz point stimulation of a two-week old cardiac patch (left) and monolayer (right); (**d**) Higher expression of desmin, alpha-actinin and cadherin in 3D-aggregates compared to in 2D-cells; (**e**) Comparison of the distribution of microtubules in 2D- and 3D-cardiac cultures. Reproduction with permission from [74,75].

Over the past decade, 3D gel culture has been widely studied to mimic the structural and functional complexity of living tissues. This method also promotes cell–cell and cell–matrix interactions, which are essential for tissue maturation [80] and provide the capability to modulate material stiffness to mimic the material properties of the heart in the developmental or disease stages [81]. Zhang et al. recently showed that hESC-derived CMs cultured in 3D fibrin hydrogels exhibit longer sarcomeres, higher conduction velocities, and enhanced mRNA expression involved in contractile function compared to the 2D culture condition (Figure 2a–c) [74]. Soares et al. cultured hiPSC-CMs in 3D spheroids (Figure 2d–e) [75]. The morphology of cardiac cells grown in 2D monolayers displayed a flattened and well spread shape. In contrast, cells grown in 3D spheroids were smaller, showed extensive cell–cell contact with several cellular junctions, and expressed higher amounts of desmin, cadherin, and alpha-actinin, which are critical components to cardiac maturation. These findings indicate that the 3D environment significantly influences cell morphology, cellular junctions, and myofibril protein expression.

It is clear that no single model (e.g., 2D or 3D cell culture methods) is likely to recapitulate all aspects of the complex genetics and biology of human diseases. In this regard, proper cell sources in combination with the 3D cell printing-based tissue modeling approach may allow the creation of patient-specific engineered 3D tissues through the localization of cells, biomolecules, and materials similar to the tissue-specific microenvironment. The most outstanding characteristic of the 3D cell printing technique is its ability to construct microscale structures consisting of physiologically relevant multiple-cellular arrangements in a single-step process. The engineered tissues in conjunction with microfluidic systems can provide an ideal test platform for use in drug discovery, analysis of chemical, biological, and toxicological agents, and basic research by offering design and system flexibility.

### 3.2. Cell Sources

The essential unit of tissue is the cell. In general, cells exhibit an intrinsic ability to self-assemble into tissue-like structures if placed in a similar geometry to that of the native tissue. Suitable exogenous factors (e.g., neighboring cells, biomaterials, and biofactors) help the cells organize a cluster, which is an essential functional unit of tissues [82]. Therefore, the supply of proper cells is a particularly important issue. Cells are typically derived from autologous tissue and autologous or allogeneic stem/progenitor cells. Stem cells possess three major properties: (a) high proliferative capacity for deriving large cell quantities; (b) paracrine factors generated by stem cells; and (c) pluripotency and directed differentiation of pluripotent cells into multiple cardiovascular lineages.

Patient-derived hPSC-derived cardiovascular cells, which can present the genetic, environmental, and physical differences of individuals, are particularly interesting. Wang et al. developed a 2D testing model of a mitochondria-related genetic disorder, Barth syndrome (BTHS), using patient iPSC-derived cardiomyocytes [83]. This model elucidated the pathophysiological phenomenon in vitro and allowed the study of the underlying disease mechanism of the cardiomyopathy of BTHS. Through this study, the researchers observed that BTHS iPSC-CMs assembled irregular sarcomeres and their contractile function was weaker than normal. Therefore, this iPSC-based tissue model provided new insight into the pathogenesis of BTHS and offered a new opportunity to find a new treatment strategy. Birey et al. generated 3D functionally integrated human forebrain spheroids using patient iPSC-derived glutamatergic and GABAergic neurons to study neural development-related diseases [84]. In this study, they demonstrated the migration of interneurons and their functional integration into human cortical ensembles, indicating the implementation of key development processes under in vitro conditions.

This new approach could help to advance our understanding of the fundamental biological and pathophysiological principles in order to improve clinical outcomes [85]. Moreover, patient-specific tissue models could be used to test therapeutic schemes and aid in the clinical diagnosis and treatment of diseases through the replacement of the injured tissues of a given patient with engineered tissues. I believe that it will lead to the discovery of innovative therapeutic methods for not just common evidence-based treatment but also patient-specific data-driven treatment.

### 3.3. Biomimicry Using Functional Bioinks

**Figure 3:**
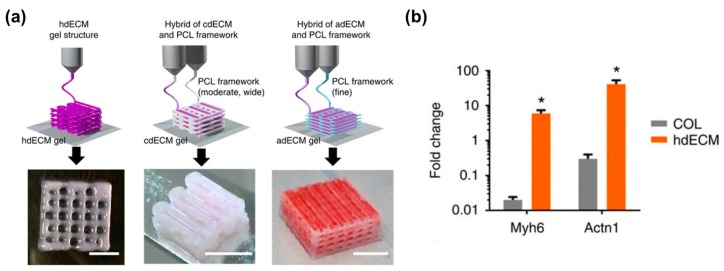

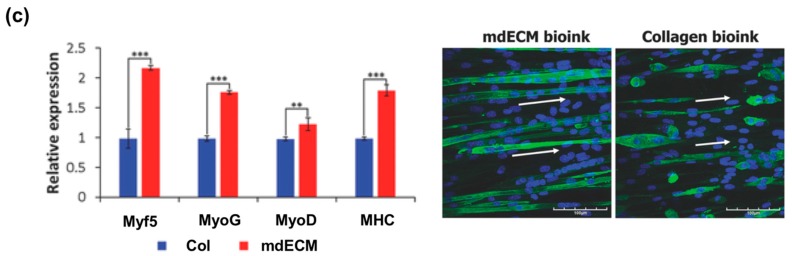
dECM bioink-based engineered tissues: (**a**) Heart tissue construct was printed with only heart dECM (hdECM). Cartilage and adipose tissues were printed with cartilage dECM (cdECM) and adipose dECM (adECM), respectively, and in combination with PCL framework (scale bar, 5 mm); (**b**) Comparative gene expression analysis for cardiogenic (Myh6 and Actn1) differentiation. All experiments were performed in triplicate. Error bars represent s.d. (* *p* < 0.05); (**c**) Gene expression of muscle constructs analyzed by real-time PCR at Day 14. (Experiments were performed with *n* = 5 per group (* *p* < 0.05, ** *p* < 0.01, and *** *p* < 0.001)), and immunofluorescence staining of myosin heavy chain (green) and nuclei (blue) in 3D cell-printed muscle construct with 40× (upper) and 60× (bottom) magnification at Day 14. White arrows indicate alignment direction. Striated muscle patterns are detected in mdECM bioink-printed muscle construct (bottom left). Scale bar in 40× image represents 100 μm, and scale bar in 60× image represents 50 μm. Reproduction with permission from [87,88].

Tissue-specific decellularized ECM (dECM) is capable of promoting cellular functions by providing a favorable constructive remodeling properties of native tissues [86]. It can recapitulate the native microenvironment of native tissues, including composition, structure, and biomechanical properties, which are critical regulators of cell fates (e.g., survival, maturation, differentiation, and migration). The polymerization mechanism of the dECM bioink is a temperature-responsible crosslinking under a physiological condition. This is a valuable feature of collagenous proteins in the dECM bioink for bioprinting purposes. Recently, a variety of tissue-derived dECMs (e.g., adipose, cartilage, cardiac muscle, skeletal muscle, and liver) have been formulated and applied as bioinks. In support of this concept, Pati et al. showed that dECM bioink induced the higher mRNA expression of cardiac-specific genes (Myh6 and Actn1) and the higher expression of the cardiac β-myosin heavy chain gene after 14 days in culture as compared with a collagen-based construct (Figure 3a,b) [87]. In addition, Choi et al. demonstrated the significantly higher expression of myogenic genes (Myf5, MyoG, MyoD, and MHC) and increased myotube formation compared with a collagen-based construct (Figure 3c) [88]. In this regard, the combination of dECM bioink with the 3D cell printing technique offers the possibility to modulate cell alignment, control graft structure, and place the inner vascular structures, which help engineered tissues achieve promoted cell activities, improve tissue functions, and accelerate therapeutic effects.

The myocardium has unique structural, mechanical, and electrical properties. In particular, the electrical coupling among the cardiomyocytes or between the cardiac and non-cardiac cells is one of the most critical factors to support their spontaneous beating function. This property provides relevant contractile forces by improving the degree of maturation, and over time, the engineered tissues can behave as normal heart muscles to a certain extent [89]. Numerous studies have shown that the composite hydrogels of mixing graphene derivatives, carbon nanotubes, or gold nanorods exhibited great effects on tissue maturation and functions [90,91,92,93]. In addition, this enhanced cell–cell signaling, cell differentiation, and their contractile forces of cardiac muscle tissues. It is expected that the electroactive materials would be a more suitable platform of in vitro drug testing by providing biomimicry of native myocardium.

### 3.4. External Stimuli

Cardiomyocytes require a long development process to ultimately reach full maturity in the adult heart. For example, hPSC-CMs could mature to adult size and morphology within 80–120 days in vitro [94]. However, this process takes a lot of effort in terms of labor, time, and cost. Thus, more strategies have been introduced to promote the maturity of hPSC-CMs, including genetic manipulation [95], modulation of microRNAs [96], delivery of biochemical factors [97], and external force stimulation [98]. Of these strategies, mechanical/electrical stimulation and hemodynamic condition are the major determinants of cardiomyocyte development, growth, and maturation [99,100].

#### 3.4.1. Mechanical Stimulation

Mechanical stimuli help with structural and functional maturation during development [101]. This can be implemented by adjusting the substrate properties such as stiffness/topography and giving tensile force. Stretching is the simplest method to deliver mechanical stimuli to hPSC-CMs-based 3D tissue models. It can be achieved by increasing the stretch over time or giving fixed distance conditions to generate automatic contractile motion. Zimmermann et al. demonstrated the effect of mechanical stress on immature cardiomyocytes seeded on collagen/matrigel hydrogel subjected to uniaxial cyclic stretch with 2 Hz frequency and 10% elongation [102]. This stimulation promoted muscle bundle organization with aligned sarcomeres and a positive inotropic response to calcium ion influx and isoproterenol [103]. Tulloch et al. generated a collagen-based 3D hPSC-CMs structure. This engineered tissue was anchored to nylon mesh tabs attached to the silicon floor of the well plate [104]. During the culture period, the engineered tissue was compacted by the tissue remodeling process and held by the nylon mesh under static tension or subjected to cyclic stretch with 1 Hz frequency and 5% elongation. After four days in culture, the results showed the upregulated expression of the β-MHC, cTnT, ANP, BNP, CACNA1C, RYR2, and SERCA2 genes. The functional characterization displayed a significant increase in active force in response to increased resting length after three weeks of culture.

Although these studies demonstrated the significance of mechanical stimulation as a maturation method for engineered heart tissues, whether the contractile forces measured from the engineered tissues were related to the type of bioink used for building tissues needs to be carefully verified. This variable could cause the misreading of the experimental outputs or the optimal parameters of mechanical stimulation protocols for the maturation of engineered tissues.

#### 3.4.2. Electrical Stimulation

**Figure 4:**
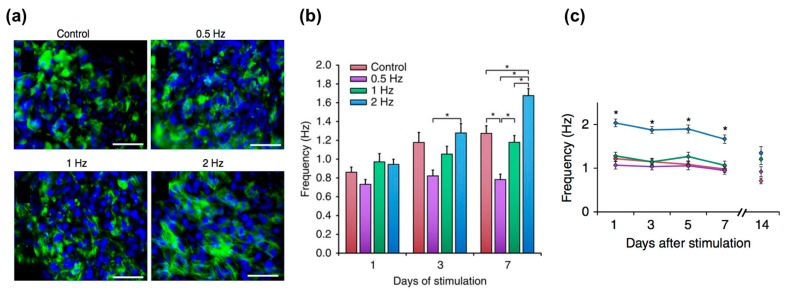
Electrical stimulation for the maturation of the engineered tissues: (**a**) Immunostaining results of troponin (green) counterstained with 4,6-diamidino-2-phenylindole (DAPI, blue). Scale bar, 50 μm; *n* ≥ 3; (**b**) Electrical stimulation for the regulation of automaticity in human stem cell-derived cardiomyocytes; (**c**) Frequency of autonomously beating cardiomyocytes as a function of duration of stimulation (average ± s.e.m., *n* = 30, * *p* < 0.05). Reproduction with permission from [22].

Electrical signal propagation is a key factor in cardiomyocytes’ contractility. The synchronous contraction of cardiomyocytes is typically propagated via the conversion of electrical signals through the coordinated activity of ion channels. Thus, the electrophysiological features, including the complex regulation of ion channels, and the electrical properties are considered the signatures of mature cardiomyocytes. Eng et al. demonstrated the capability of electrical conditioning to promote embryonic body differentiation from hPSCs (Figure 4) [22]. These 3D spheroid-shaped engineered tissues were subjected to electrical stimulation of 1–2 Hz frequency, 2 ms pulse, and 5 V/cm intensity continuously for seven days. This stimulation promoted junction protein expression (e.g., connexin) and sarcomeric formation. In addition, the engineered tissues adapted their beating rate to the given frequency at which they were stimulated and maintained the adapted beating rates for up to two weeks after the end of stimulation. Ruan et al. created engineered heart tissues using hiPSC-CMs mixed with collagen hydrogel and then cultured them under the stress or combined static stress and electrical pacing conditions [98]. The engineered tissues were cultured for a week of static stress and then subjected to electrical stimulation of 2 Hz frequency, 5 ms pulse, and 5 V/cm intensity continuously for another week. This combinatorial stimulation enhanced the cell alignment, passive stiffness, cardiac hypertrophy, and contractility of the engineered tissues as well as the expression of SR-related proteins, such as RYR2 and SERCA2.

#### 3.4.3. Integration with Microfluidics

**Figure 5:**
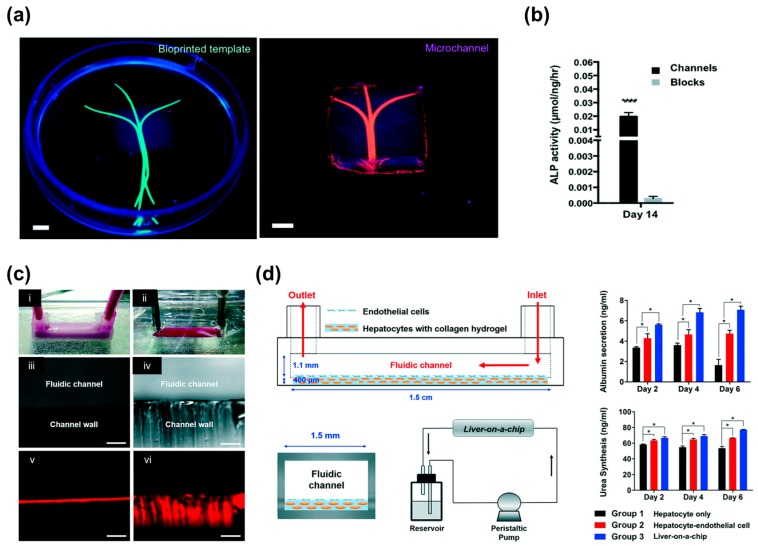
3D printed microfluidic models: (**a**) Photographs of the bioprinted templates (green) enclosed in GelMA hydrogels and the respective microchannels perfused with a fluorescent microbead suspension (pink); (**b**) Significantly higher ALP activity levels in cell-laden constructs with microchannels versus cell-laden hydrogel blocks on Day 14 (**** *p* < 0.0001). (Scale bar, 700 μm); (**c**) Channels made by the: (i) PCL material; and (ii) PDMS material; (**d**) Schematic illustration of the side view, vertical section view, and perfusion system of the liver-on-a-chip. Liver function analysis with albumin and urea tests (* *p* < 0.05). Reproduction with permission from [113,114].

3D bioprinting has been mainly utilized only for fabricating tissue constructs (e.g., skin, bone, blood vessels, liver, heart tissue, and cartilage tissue) [38,50,105,106,107,108,109,110,111]; however, there is huge potential to integrate microfluidic systems and 3D printed tissue models because of the process flexibility offered by multi-materials. In addition, this integrated system would enable the elucidation of the physiological phenomena (e.g., interactions between immune cells/blood and tissues) on the 3D tissue models that occur in our body system. The high-throughput 3D tissue fabrication process could result in the development of “organ-on-chips” for biological research, drug screening, and toxicology [112]. The concept of organ-on-chips can provide the basis for preclinical assays of new drugs with great prediction capability. However, the multi-step and complicated chip fabrication processes, such as PDMS polymerization, chip bonding, and secondary cell seeding, make it difficult to provide consistent production yields and physiologically relevant environments (e.g., 3D cell–cell or cell–matrix interactions) for spatial heterogeneity similar to that found in the native tissues [113]. In this sense, 3D bioprinting can produce 3D cellular arrangements and ECM microenvironments as well as microfluidic channels in a one-step fabrication process. Recently, Bertassoni et al. developed 3D tissue models with perfusable vascular channels using 3D bioprinting of the agarose bioink and the hydrogel molding method. The agarose channel was removed after the polymerization molding materials (cell-laden GelMA hydrogels), and the fabricated microchannels promoted the mass transport, viability, and differentiation of the pre-osteoblast cell lines (MC3T3 cells) embedded in the GelMA hydrogels (Figure 5a,b) [114]. Lee et al. developed a 3D bioprinted liver-on-a-chip platform using one-step fabrication [113]. To create a microfluidic device, they used PCL to generate a microfluidic device and then placed the hepatocyte cell line (HepG2) and human umbilical vein endothelial cells (HUVECs) embedded in each collagen bioink into the inner chamber of the device. This device had lower protein absorption properties compared to the polydimethylsiloxane (PDMS) platform, indicating that it possessed the capability to accurately measure cell metabolism and drug sensitivity (Figure 5c). The integration of a vascular network with engineered cardiovascular tissues has been shown to increase cell viability and functionality (Figure 5d) [59,113,115,116]. Thus, a 3D bioprinted biomimetic tissue structure in conjunction with a microfluidic system is more likely to provide the actual organ-level response.

## 4. Vascularization of Cardiovascular Tissues

Perfusable channels enable the creation of vascular networks in 3D tissues and promote rapid vascularization, survival, and functions. In general, the human microvasculature is consecutively divided into small branches, and each has a different role in defining the function of the vascular network. For example, metarterioles (80–100 μm) serve as a vascular shunt to redistribute blood and nutrients. These microvessels usually form a thoroughfare channel for a perfusable network to allow the efficient exchange of metabolites [117]. There have been several approaches to 3D tissue modeling for cardiovascular diseases [118,119], and new findings are continuously reported in the field of 3D bioprinting-based tissue engineering. Cardiovascular diseases are particularly correlated with the perfusion of oxygen and nutrients through the integrated channel (vascular network). Hence, advanced vascularization techniques are required to create physiologically functional tissues.

The lack of control over the organization of the vasculature hampers the function of the constructs. To overcome this limitation, recent studies have suggested the incorporation of cells or biofactors in the engineered tissues, which can accelerate the vascularization of the implanted construct and improve the long-term tissue survival [33,34,120,121]. The mixture of vascular cells in the structure forms randomly distributed capillary-like vessels that conduct blood flow very slowly with limited functional enhancement [38]. However, it is difficult to recapitulate the vasculature of organs that are comprised of multiple cells and a complex network. Thus, the geometric control of the vascular plexus promotes rapid arteriogenesis and enhances blood supply as well. In addition, the highly organized vascular network can be successfully connected to the patient’s vasculature [38,122].

### 4.1. Perfusable Microchannels: Use of a Sacrificial Material

**Figure 6:**
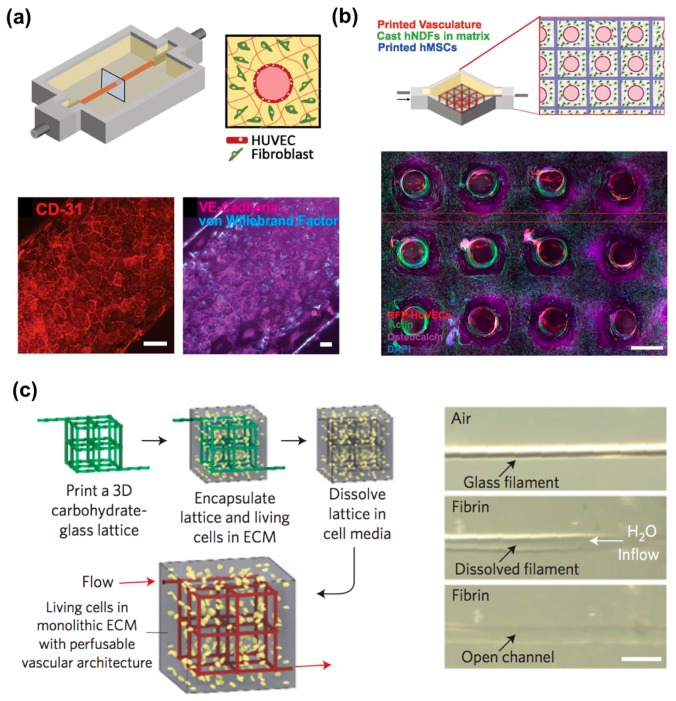
3D vascularized tissues remain stable during long-term perfusion: (**a**) Schematic depicting a single HUVEC-lined vascular channel supporting a fibroblast cell-laden matrix and housed within a 3D perfusion chip; (**b**) Confocal microscopy image through a cross-section of 1-cm-thick vascularized osteogenic tissue construct after 30 d of active perfusion and in situ differentiation. (Scale bar, 1.5 mm); (**c**) A vascular network via the rapid casting of printed sacrificial bioglass. Reproduction with permission from [59,116].

There are two main strategies to forming macrovasculature using 3D bioprinting technology. One is the use of a sacrificial material (e.g., Pluronic F127, agarose, gelatin, glass), leaving a vascular network behind [59,114,116,123,124,125,126]. Kolesky et al. fabricated a thick (e.g., over 1 cm) engineered bone tissue composed of fibrin-gelatin blended hydrogels and multiple cell types (Figure 6a,b) [59]. The mold was fabricated using elastomeric ink (PDMS), and the fugitive ink (a blend of Pluronic F127 and thrombin) printed the vascular network. Next, the cell-laden ink, composed of fibrinogen, gelatin, thrombin, and transglutaminase, was cast into the prefabricated mold. After casting, the fugitive ink was perfused out of the construct, and then endothelial cells were seeded to line the hollow tube for vascularization. This 3D vascularized tissue was cultured with the perfusion of growth factors for over six weeks to promote in situ bone formation. However, this technique can make a relatively simplified tissue architecture that is made of homogenous cell-laden matrices of natural tissues composed of heterogeneous cell sources and complex designs. Miller et al. generated a vascular network via the rapid casting of printed sacrificial Bioglass, which can be dissolved by water (Figure 6c) [116]. After printing the 3D filament vascular network, they coated it with biopolymer and then cast it into a 3D ECM hydrogel. Endothelial cells were then lined inside the engineered lumen by perfusion seeding, and finally, the engineered tissues showed sustained metabolic function of primary rat hepatocytes.

### 4.2. Perfusable Microchannels: Tube-Like Structures

**Figure 7:**
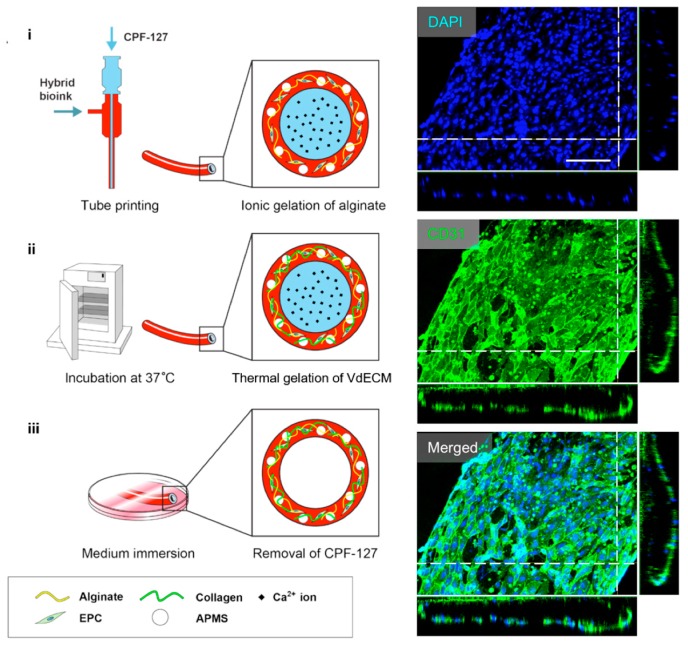
A schematic depiction of the bio-blood vessel (BBV) fabrication process. The ionic gelation of alginate in the bioink realized BBV printing. CD31/DAPI staining indicated that the encapsulated EPCs formed a layer of fully differentiated endothelium on the BBV after culturing for seven days. Reproduction with permission from [127].

By applying the particularly designed nozzles such as multi-axial nozzles, a tube-like structure can be created directly after the fabrication process. Zhang et al. fabricated a vessel-like microfluidic channel using a co-axial nozzle system that enabled the direct printing of a hollow vascular channel in a 3D hydrogel [126]. Gao et al. also developed a tube-like structure using a co-axial bioprinting technique [127]. Interestingly, they used a hybrid bioink composed of dECM derived from aortic tissue and sodium alginate hydrogel and mixed with endothelial progenitor cells, and this hybrid bioink was supplemented with microcapsules of the atorvastatin drug (Figure 7). The results showed striking performance in promoting survival and vascularization potential both in vitro and in vivo. In addition, the engineered blood vessel maintained patency and formed a fully differentiated endothelial layer after culture for seven days in vitro.

### 4.3. Self-Assembly

**Figure 8:**
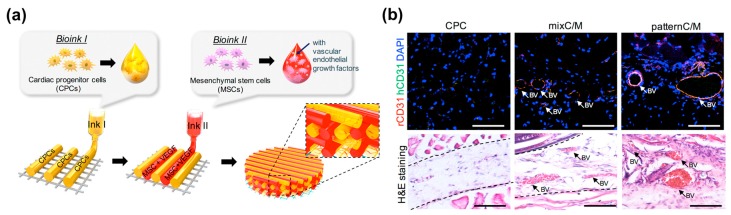
3D printing of self-assembled cardiovascular tissues: (**a**) Illustration of pre-vascularized stem cell patch including multiple cell-laden bioinks and supporting PCL polymer; (**b**) Effects of pre-vascularized stem cell patch on the vascular formation in vivo: Histological analysis and immunofluorescence staining results of the experimental groups four weeks after implantation (Scale bar, 200 μm; BV = blood vessel). Reproduction with permission from [38].

This strategy is the printing of vascular cells directly in the engineered tissues [38,118,126,128]. ECM remodeling allows vascular tissue assembly, leading to the fusion and maturation of scaffold-free tissues around the endothelial cells as well as the tight fusion of the tissues to the engineered vasculature [129]. This macrovasculature creation is also expected to form the surrounding capillaries by the natural vascular remodeling process. In addition, the direct printing of vascular networks possesses a high scalability that facilitates the programmable arrangement of cellular components with a desired architecture. Jang et al. demonstrated that the multicellular patterning of cardiac patches promotes the survival of delivered stem cells (Figure 8a,b) [38]. The patterned vascular cells (human turbinate tissue-derived mesenchymal stem cells supplemented with VEGF) promoted rapid host-graft anastomosis and tissue formation. It also ameliorated cardiac function and cellular infiltration into the infarct area as well as reduced cardiac hypertrophy and fibrosis. Ma et al. fabricated a 3D hexagonal liver lobule tissue composed of hepatocytes and supporting cells [115]. They used a digital micro-mirror device (DMD) system that uses LED light (365 nm) for the polymerization of light-active bioink. Human-induced pluripotent stem cell-derived hepatic progenitor cells (hiPSCs-HPCs) were encapsulated in GelMA bioink and printed with a hexagonal shape. The supporting cells (i.e., HUVECs and human ASCs) were embedded in the mixture of glycidyl methacrylated-hyaluronic acid (GMHA) and placed in between HPC-laden hexagonal shapes to promote vascularization. As a result, this engineered tissue improved the expression of liver-specific marker genes as well as the secretion of metabolic products (e.g., urea and albumin) while maintaining the printed cellular pattern of the liver lobule.

## 5. Future Perspectives and Concluding Remark

Recent advancements in 3D bioprinting and tissue engineering have shown remarkable capability to provide platforms for studying of unknown mechanism of development, intractable diseases, and responsiveness of newly developed drugs. This technique continues to grow and evolve with the addition of various outstanding technologies, such as bioelectronics, next-generation sequencing, and genetic engineering [130,131]. In particular, the convergence of electronics and 3D bioprinting helps to create functional living tissues, such as engineered skin with tactile sensitivity, ears capable of hearing, and cardiac muscles with electrophysiological signals [130,132]. Furthermore, this integration of biosensors and engineered tissue devices facilitates real-time monitoring, which can provide new insights into tissue morphogenesis, pathogenesis, and drug-responsive remodeling processes. These systems can potentially shift the paradigm toward creating living sensor and actuator systems that are impossible to achieve using the traditional manufacturing and electrical engineering approaches. Personalized tissue models would also be an interesting application of the 3D bioprinting platform. With the convergence of patient-derived stem cell engineering, 3D printed tissues can reflect the in vivo responses of individual characteristics. Thus, this platform facilitates a higher treatment effect by enabling the selection of appropriate types of drugs, but the usage amount is less than that of the conventional approach. Moreover, medical information, including information on genomes, proteins, and medical/family history, could be directly applied to recapitulate personalized pathophysiological conditions in vitro to aid in the clinical diagnosis and treatment of diseases [131].
